# Systematic Review of Genotype-Phenotype Correlations in Frasier Syndrome

**DOI:** 10.1016/j.ekir.2021.07.010

**Published:** 2021-07-16

**Authors:** Yurika Tsuji, Tomohiko Yamamura, China Nagano, Tomoko Horinouchi, Nana Sakakibara, Shinya Ishiko, Yuya Aoto, Rini Rossanti, Eri Okada, Eriko Tanaka, Koji Tsugawa, Takayuki Okamoto, Toshihiro Sawai, Yoshinori Araki, Yuko Shima, Koichi Nakanishi, Hiroaki Nagase, Masafumi Matsuo, Kazumoto Iijima, Kandai Nozu

**Affiliations:** 1 Department of Pediatrics, Kobe University Graduate School of Medicine, Kobe, Japan; 2 Department of Pediatrics, Kyorin University School of Medicine, Mitaka, Japan; 3 Department of Pediatrics, Hirosaki University Hospital, Hirosaki, Japan; 4 Department of Pediatrics, Hokkaido University Graduate School of Meidicine, Sapporo, Japan; 5 Department of Pediatrics, Shiga University of Medical Science, Shiga, Japan; 6 Department of Pediatrics, Hokkaido Medical Center, Sapporo, Japan; 7 Department of Pediatrics, Wakayama Medical University, Wakayama, Japan; 8 Department of Child Health and Welfare (Pediatrics), Graduate School of Medicine, University of the Ryukyus, Okinawa, Japan; 9 Locomotion Biology Research Center, Kobe Gakuin University, Kobe, Japan

**Keywords:** genotype-phenotype correlation, Frasier syndrome, minigene assay, systematic review, transcript analysis

## Abstract

**Introduction:**

Frasier syndrome (FS) is a rare inherited kidney disease caused by intron 9 splicing variants of *WT1*. For wild-type *WT1*, 2 active splice donor sites in intron 9 cause a mixture of 2 essential transcripts (with or without lysine-threonine-serine [+/KTS or −KTS]), and imbalance of the +KTS/−KTS ratio results in the development of FS. To date, 6 causative intron 9 variants have been identified; however, detailed transcript analysis has not yet been conducted and the genotype-phenotype correlation also remains to be elucidated.

**Methods:**

We conducted an *in vitro* minigene splicing assay for 6 reported causative variants and *in vivo* RNA sequencing to determine the +KTS/−KTS ratio using patients’ samples. We also performed a systematic review of reported FS cases with a description of the renal phenotype.

**Results:**

The *in vitro* assay revealed that although all mutant alleles produced −KTS transcripts only, the wild-type allele produced both +KTS and −KTS transcripts at a 1:1 ratio. *In vivo* RNA sequencing showed that patients’ samples with all heterozygous variants produced similar ratios of +KTS to −KTS (1:3.2−1:3.5) and wild-type kidney showed almost a 1:1 ratio (1:0.85). A systematic review of 126 cases clarified that the median age of developing ESKD was 16 years in all FS patients, and there were no statistically significant differences between the genotypes or sex chromosome karyotypes in terms of the renal survival period.

**Conclusion:**

Our study suggested no differences in splicing pattern or renal survival period among reported intron 9 variants causative of FS.

## Graphical abstract

**Figure:**
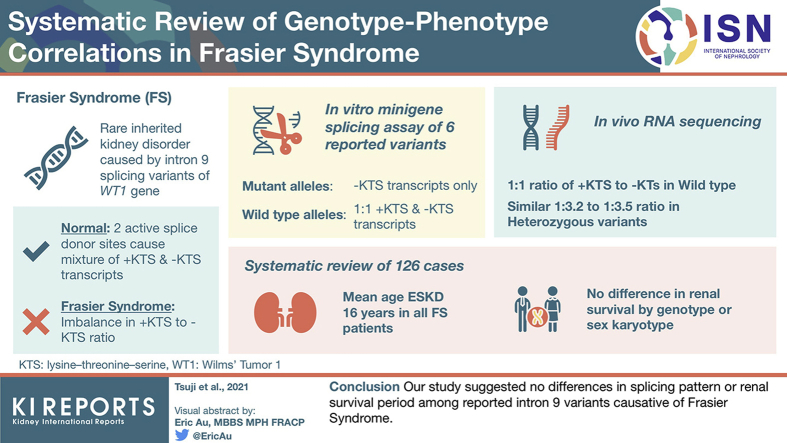

Frasier syndrome is a rare inherited disease characterized by steroid-resistant nephrotic syndrome with proteinuria that begins in early childhood and progressively worsens with age. It is also associated with a high risk of developing ESKD, gonadal tumors, and male pseudohermaphroditism (female external genitalia with XY sex chromosomes). It is caused by splice donor site variants in intron 9 of the Wilms’ tumor 1 (*WT1* [NM_024426.6]) gene, with 6 variants identified thus far (c.1432+1 G>A, c.1432+2 T>C, c.1432+4 C>T, c.1432+5 G>A, c.1432+5 G>T, and c.1432+6 T>A).^1^, ^2^, ^3^, ^4^ *WT1* is composed of 10 exons and encodes a transcription factor with 4 zinc finger motifs; this transcription factor plays an important role in early gonadal and renal development by regulating the sex-determining region Y.^5^^,^^6^ Two active splice donor sites of intron 9 result in a mixture of 2 transcripts with or without 3 amino acids (+KTS or −KTS) between zinc fingers 3 and 4.^7^

Although the roles of +KTS and −KTS isoforms have not yet been completely clarified, it has been proposed that the −KTS isoform may function mostly as a transcriptional regulator, whereas the +KTS isoform acts predominantly at the posttranscriptional level.^8^^,^^9^ Furthermore, studies in animal models have also shown that these isoforms are essential for survival. Hammes *et al.*^10^ introduced the heterozygous variant into intron 9 of *Wt1* in mice using a Cre-loxP strategy. These model mice showed a reduction of +KTS level and developed glomerulosclerosis, representing a model of FS. In contrast, the mice with homozygosity of this variant showed a complete lack of the +KTS isoform and died within 24 hours after birth due to kidney defects.^10^

It is reported that the normal range of the WT1 +/−KTS isoform ratio range is between approximately 5:2 and 1:1 (average 3:2), whereas intron 9 variants lead to a shift in this ratio to between 1:2 and 1:5 (average 1:2) in FS (Table 1),^3^ which indicates marked variability. However, all of these studies quantified the transcripts using a semiquantitative method based on agarose gel electrophoresis and need to be reexamined by quantitative analysis using RNA sequencing. Furthermore, *in vitro* assays on the role of intron 9 variants have been performed in 3 studies; however, these studies were limited to only 4 of the 6 known variants (c.1432+2 T>C, c.1432+4 C>T, c.1432+5 G>A, and c.1432+5 G>T).^2^^,^^3^^,^^11^^,^^12^

**Table 1:** +KTS/−KTS ratio determined by transcript analysis in previous studies and our targeted RNA sequencing

Variant	Tissue or cell	Average read depth			Reference
		+KTS	−KTS	+KTS/−KTS ratio	
Our study					
c.1432+3 G>T	Urine-derived cell	363	1276	1:3.5	—
c.1432+4 C>T	Urine-derived cell	136	430	1:3.2	—
c.1432+5 G>A	Kidney	117	407	1:3.5	—
Normal	Kidney	213	181	1:0.85	—
Previous study					
c.1432+4 C>T	Gonadal tissue	—	—	1:3.1–4.8	^45^
	Lymphocyte	—	—	1:2.3–2.6	^3^^,^^27^
c.1432+5 G>A	Gonadal tissue	—	—	1:1.8–1.9	^4^
	Lymphoblastoid cell	—	—	1:2.5	^11^
	Kidney and ovary	—	—	1:2.9	^11^
c.1432+6 T>A	Lymphocyte	—	—	1:2.1	^3^
Normal	Epididymis	—	—	1:0.29–0.59	^45^
	Kidney	—	—	1:0.42	^4^
	Lymphoblastoid cell	—	—	1:0.67	^11^
	Lymphocyte	—	—	1:0.67–0.83	^3^^,^^27^

+KTS, with lysine-threonine-serine; −KTS, without lysine-threonine-serine.

Here, we conducted an *in vitro* splicing assay to measure the +/−KTS ratio for all reported intronic variants (c.1432+1 G>A, c.1432+2 T>C, c.1432+4 C>T, c.1432+5 G>A, c.1432+5 G>T, and c.1432+6 T>A) and a variant newly identified in our study (c.1432+3 G>T). In addition, *in vivo* RNA sequencing using patients’ kidney-derived samples was conducted for 2 major variants (c.1432+4 C>T, c.1432+5 G>A) and a newly identified variant (c.1432+3 G>T). Furthermore, we conducted a systematic review of previously reported cases of FS. With these analyses, we aimed to uncover genotype-phenotype correlations in FS and to determine differences in splicing pattern and clinical phenotype, mainly renal survival period, among the variants.

## Methods

### *In Vitro* Splicing Assay

For *in vitro* splicing experiments, hybrid minigene constructs were created by inserting a test sequence comprising exon 9 of *WT1* and its flanking introns into a multiple cloning site within the intron between exons A and B of the minigene construct (H492) built into the pcDNA 3.0 mammalian expression vector (Thermo Fisher Scientific, Waltham, MA) as reported previously^13^^,^^14^ (Supplementary Figure S1). The test sequence was obtained by amplifying the control sample by polymerase chain reaction using primers that corresponded to introns 8 and 9 (Supplementary Table S1). These primers included NheI and BamHI restriction enzyme recognition sites, respectively. The amplified products were digested with NheI and BamHI (New England Biolabs, Ipswich, MA) and inserted into the minigene that had been digested with the same restriction enzymes. Furthermore, constructs containing each of the reported intronic variants were generated by mutagenesis (the primers used are listed in Supplementary Table S2).

Using this method, we constructed both wild-type and mutant hybrid minigenes that carried exon 9 and either the wild-type or a mutated (c.1432+1 G>A, c.1432+2 T>C, c.1432+3 G>T, c.1432+4 C>T, c.1432+5 G>A, c.1432+5 G>T, and c.1432+6 T>A) intron 9 sequence. After the construct sequences had been confirmed by Sanger sequencing, the hybrid minigenes were transfected into HeLa and HEK293T cells with Lipofectamine 3000 (Thermo Fisher Scientific). These 2 cell types were chosen because both of them showed high efficiency for *in vitro* transfection and expressed sufficient levels of transfected mRNA in previous analysis for genes expressed predominantly in the kidney.^15^^,^^16^ Cells were harvested 24 hours after transfection, and total RNA was extracted with a RNeasy Plus Mini Kit (Qiagen, Hilden, Germany). One microgram of total RNA was subjected to reverse transcription with the RNA to cDNA EcoDry Premix (Doubled Primed; Takara Bio, Otsu, Japan), and polymerase chain reaction was subsequently performed using a forward primer corresponding to a segment of the upstream exon A and a reverse primer complementary to a segment of the downstream exon B as previously described.^13^ Polymerase chain reaction products were analyzed with the Agilent 2100 Bioanalyzer (Agilent Technologies, Santa Clara, CA) and direct sequencing (3130 Genetic Analyzer, Thermo Fisher Scientific).

### *In Vivo* mRNA Analysis (Targeted RNA Sequencing)

To clarify the +KTS/−KTS mRNA ratio for *in vivo* samples, we analyzed patients’ samples for the most common variants (c.1432+4 C>T and c.1432+5 G>A). Furthermore, we analyzed the sample with the novel variant c.1432+3 G>T to confirm splicing abnormality. Total RNA was extracted from urine-derived cells (+3, +4) and kidney (+5) using the RNeasy Plus Mini Kit and ISOGEN RNA extraction reagent (Nippon Gene Co., Toyama, Japan), respectively. Samples of other variants were not available because of their rarity. We also obtained the wild-type kidney total RNA (Human Kidney Total RNA, #636529; Clontech Laboratories Inc., Mountain View, CA). A custom panel to detect *WT1* was designed for the targeted RNA sequences. Targeted RNA sequencing samples were prepared using a SureSelect XT RNA Direct Reagent Kit (Agilent Technologies) in accordance with the manufacturer’s instructions. Amplified target libraries were then sequenced via MiSeq (Illumina, San Diego, CA). Trimmed and filtered reads were aligned to the human reference genome and transcriptome UCSC hg19 using Strand NGS 3.4 software (Strand Life Science Pvt. Ltd., Bangalore, India). +KTS/−KTS ratios were calculated from the average read depth in the last 9 bases of exon 9 for each +KTS/−KTS mRNA (Supplementary Figure S1).

### Systematic Review

A systematic search of the PubMed database (https://www.ncbi.nlm.nih.gov/pubmed/) was performed, and only those studies published in English and before December 2020 were included. The following search terms were used: “Frasier syndrome” or “Nephrotic syndrome AND WT1.” An additional search was conducted by selecting articles that included genotype and clinical data relevant to the current study, such as age of onset of nephrotic syndrome or ESKD, sex chromosomes, external genitalia, or tumor, from the reference sections of review articles. We constructed renal survival curves by Kaplan-Meier analysis to determine the prognosis and median age of onset of ESKD for each variant, and we determined that as the median age of onset of ESKD became younger, it increased in severity. Furthermore, we summarized the phenotype data reported for each variant including clinical descriptions relating to the external genitalia and sex chromosomes. In this study, patients were classified into the following 3 groups depending on their sex chromosome karyotype and the presence of disorder of sexual development (DSD): XX female (all patients with XX karyotype), XY female (XY karyotype with DSD), and XY male (XY karyotype without DSD). The definition of nephrotic syndrome in this study followed the description of each case report, and many of those reports did not include the detailed definition of nephrotic syndrome. Gonadal abnormality was defined as a mismatch between the sex of the chromosomes and the external genital phenotype.

### Statistical Analysis

All calculations were performed using standard statistical software (JMP for Mac, version 14; SAS Institute, Cary, NC). The occurrence of events (renal survival period) was examined using the Kaplan-Meier and log-rank tests. Associations were considered to be statistically significant when *P* values were < 0.05.

## Results

### *In Vitro* Splicing Assay

**Figure 1:**
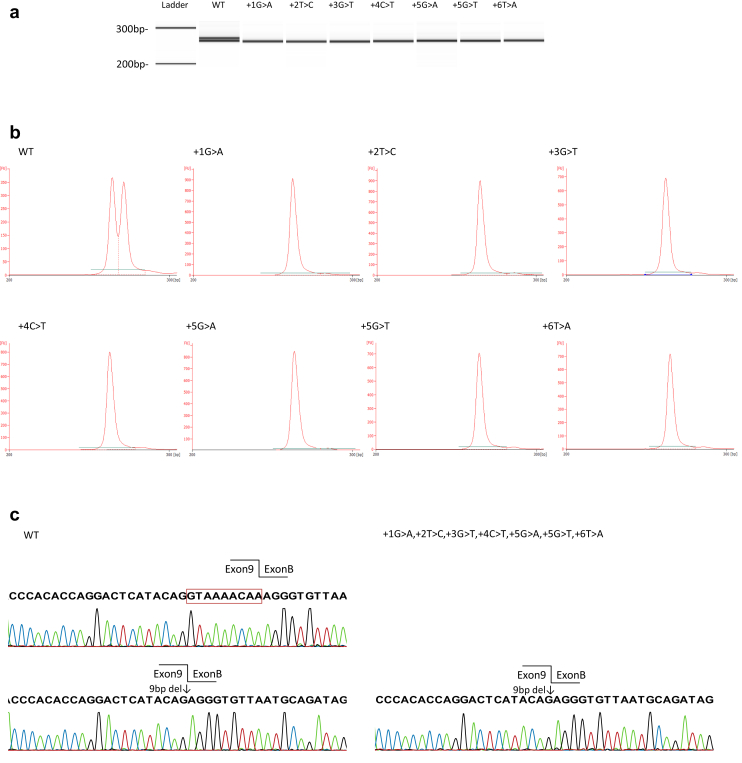
Reverse transcription polymerase chain reaction products of the intron 9 wild-type and mutant hybrid minigene transcripts. (a) Electrophoresis of the reverse transcription polymerase chain reaction amplicons with a bioanalyzer showed that the wild-type minigene construct produced 2 bands, whereas the mutant constructs only produced 1 band that was similar in size to the smaller wild-type band. This *in vitro* assay was performed in HEK293T cell lines. (b) Semiquantitative analysis of the amplicons with a bioanalyzer indicated that the ratio of the wild-type products was 1:1. All of the mutant vectors produced only 1 band of the same size. (c) Sanger sequencing of the amplicons showed that both with (+KTS) and without lysine-threonine-serine (−KTS) sequences were produced by the wild-type vector, whereas only the −KTS sequence was obtained from all of the mutant vectors.

We conducted an *in vitro* splicing assay of wild-type and mutant minigene constructs to determine the splicing patterns resulting from the 6 intron 9 variants reported to date and a variant that we discovered (c.1432+3 G>T) associated with FS. Polymerase chain reaction products from the wild-type construct produced 2 bands following capillary electrophoresis (Figure 1a). Semiquantitative analysis with a bioanalyzer revealed that the molecular ratio of these 2 bands was approximately 1:1 (39.6 nmol/l:40.5 nmol/l). Sequencing of the amplicons from the wild-type construct indicated the presence of both +KTS and −KTS transcripts, whereas all mutant constructs produced only −KTS transcripts (Figure 1).

### *In Vivo* mRNA Analysis

We conducted an *in vivo* mRNA analysis for 3 variants that were available for analysis: 2 variants from urine-derived cells (heterozygotes of 1432+3 G>T and c.1432 +4 C>T) and 1 from a kidney biopsy sample (heterozygote of c.1432 +5G>A), along with the wild-type kidney sample. The average depth of sequencing in the target region ranged from 394 to 1639 (Table 1). Our analysis revealed that the +KTS/−KTS ratio was 1:0.85 in a wild-type human kidney but 1:3.2 in c.1432+4 C>T and 1:3.5 in c.1432+3 G>T and c.1432+5 G>A (Table 1, Supplementary Figure S1). Although statistical analysis could not be conducted because only a single sample from each variant was available for analysis, all analyzed variants including the newly detected variant c.1432+3 G>T showed almost the same +/−KTS ratio in RNA sequencing.

### Systematic Review

Genetic and clinical data were obtained from a total of 126 cases: 115 cases described in previous reports^6^^,^^11^^,^^12^^,^^17^, ^18^, ^19^, ^20^, ^21^, ^22^, ^23^, ^24^, ^25^, ^26^, ^27^, ^28^, ^29^, ^30^, ^31^, ^32^, ^33^, ^34^, ^35^, ^36^, ^37^, ^38^, ^39^, ^40^, ^41^, ^42^, ^43^, ^44^, ^45^, ^46^, ^47^, ^48^, ^49^, ^50^, ^51^, ^52^, ^53^, ^54^, ^55^, ^56^, ^57^, ^58^ and 11 cases diagnosed at our institution (Table 2). There were no significant differences in the proportions of patients with female external genitalia and XY chromosomes between the 2 major variants: c.1432+4 C>T (44/49, 90%) and c.1432+5 G>A (30/35, 86%) (Table 2, *P* = 0.57, χ^2^ test). We could not conduct statistical analysis for the other variants because the sample numbers were too low. Regarding tumorigenesis, 1 and 31 cases had a Wilms tumor and a gonadal tumor, respectively. In addition, 14 cases had undergone prophylactic gonadectomy. However, descriptions of a Wilms tumor or a gonadal tumor were only provided in 30 and 65 cases, respectively, so the prevalence of tumor in this study was calculated as 3.3% (1/30 cases) for a Wilms tumor and 61% (31/51 cases without prophylactic gonadectomy) for a gonadal tumor. We also analyzed the tumorigenesis for each sex chromosome karyotype and external genitalia (Supplementary Table S2). The results showed that 85% of patients who were XY female and none with XX chromosomes developed a gonadal tumor. Interestingly, 40% (2 in 5) of patients who were XY male developed a gonadal tumor, both of whom had been diagnosed with bilateral cryptorchidism.^36^^,^^45^

**Table 2:** Clinical descriptions of the external genitalia, sex chromosomes, renal manifestations, and gonadal/Wilms tumor

*WT1* mutations	*N* with data^a^	Sex chromosomes and external genitalia			Renal manifestations		Gonadal tumor		Wilms tumor
		XX female	XY female	XY male	Median age of developing nephrotic syndrome (95% CI)	Median age of developing ESRD (95% CI)	Tumor detected	Prophylactic gonadectomy	Tumor detected
c.1432+1G>A	3	1	2	0	2 (n/c)	7.5 (6–9)	1	0	0
c.1432+2T>C	2	0	2	0	5.5 (5–6)	23 (n/c)	0	0	0
c.1432+3G>T	1	1	0	0	3 (n/c)	n/c	0	0	0
c.1432+4C>T	66^a^	14	44	5	4 (3–5)	17 (13–22)	19	8	1
c.1432+5G>A	51^a^	15	30	5	4 (2–5)	15 (10–19)	11	4	0
c.1432+5G>T	2	0	2	0	3 (n/c)	25 (n/c)	0	1	0
c.1432+6T>A	1	0	1	0	2 (n/c)	35 (n/c)	0	1	0
All	126	31	81	10	4 (3–5)	16 (14–22)	31	14	1

CI, confidence interval; ESRD, end-stage renal disease.

a Sex chromosome karyotypes were not described in 3 cases of c.1432+4C>T and 1 case of c.1432+5G>A.

**Figure 2:**
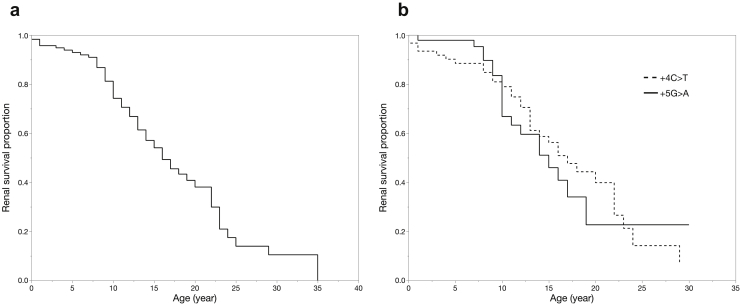
The renal survival rates associated with the intron 9 variants. (a) The renal survival curve for all variants (*n* = 116); the median age for the development of end-stage kidney disease (ESKD) was 16 years. (b) The renal survival curve for 2 major variants. The dashed line indicates patients with +4 C>T (*n* = 61); the median age for the development of ESKD was 17 years in these patients. The solid line indicates patients with +5 G>A (*n* = 47); the median age for the development of ESKD was 15 years in these patients. There was no significant difference between the 2 groups (*P* = 0.62, log-rank test).

**Figure 3:**
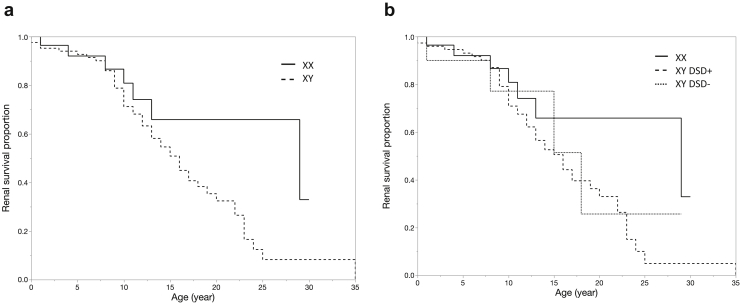
The renal survival rates based on sex chromosomes and sexual development. (a) The renal survival curves for each sex chromosome karyotype. The solid line indicates patients with XX chromosomes (*n* = 28); the median age for the development end-stage kidney disease (ESKD) was 29 years in these patients. The dashed line indicates patients with XY chromosomes (*n* = 84); the median age for the development of ESKD was 16 years in these patients. There was no significant difference between these 2 groups (*P* = 0.09, log-rank test). (b) The renal survival curves for the following groups: group 1, patients with XX sex chromosomes; group 2, patients with XY sex chromosomes and disorder of sexual development (DSD); and group 3, patients with XY sex chromosomes without DSD. The solid line indicates group 1 (*n* = 28); the median age for the development of ESKD was 29 years in these patients. The dashed line indicates group 2 (*n* = 74); the median age for the development of ESKD was 16 years in these patients. The dotted line indicates group 3 (*n* = 10); the median age for the development of ESKD was 18 years in these patients. There were no significant differences among these groups.

Regarding renal symptoms, among the 126 cases, there were descriptions of the age of onset of obvious proteinuria or nephrotic syndrome and kidney function for 102 and 116 cases, respectively. The results showed that the median age of onset of proteinuria was 4 years and that 58 cases had progressed to ESKD. We also created renal survival curves for all variants as well as individually for 2 major intronic variants (c.1432+4 C>T and c.1432+5 G>A) using Kaplan-Meier analysis (c.1432+1 G>A, *n* = 2; c.1432+2 T>C, *n* = 2; c.1432+3G>T, *n* = 1; c.1432+4 C>T, *n* = 61; c.1432+5 G>A, *n* = 47; c.1432+5 G>T, *n* = 2; and c.1432+6 T>A, *n* = 1) (Figure 2a and b). This analysis revealed that the median age of developing ESKD in the whole cohort was 16 years old. There was no significant difference between the 2 major variants c.1432+4 C>T (*n* = 61) and c.1432+5 G>A (*n* = 47) in the age of ESKD onset (17 vs. 15 years, *P* = 0.62, log-rank test; Figure 3a).

We divided the patients according to their sex chromosome karyotypes and analyzed the groups for differences in renal prognosis. No significant difference in the age of ESKD onset was found between the patients with XX chromosomes (*n* = 28) and XY chromosomes (*n* = 84) (29 vs. 16 years, *P* = 0.09, log-rank test). In addition, to analyze whether the presence of DSD had any effect on renal prognosis, we classified the patients into the following 3 groups: group 1, XX (*n* = 28); group 2, XY with DSD (*n* = 74); and group 3, XY without DSD (*n* = 10). However, there were no significant differences in the renal survival period among these groups (Figure 3b).

## Discussion

In this study, we conducted *in vitro* and *in vivo* splicing assays for all reported and newly identified variants in intron 9 of *WT1* resulting in FS. In addition, we examined the genetic and clinical characteristics of reported patients with FS via a systematic review. Our results confirmed the presence of an abnormal splicing pattern in all variants both *in vitro* and *in vivo*. In addition, +KTS/−KTS mRNA ratios were equal for all variants evaluated in both analyses. Furthermore, a systematic review showed that there was no difference in renal prognosis according to genotype. Therefore, it was suggested that genotype does not affect renal prognosis in FS caused by *WT1* intron 9 variants.

In previous studies, transcript analysis was only performed for 4 of the intron 9 variants associated with FS: c.1432+2 T>C, c.1432+4 C>T, c.1432+5 G>A, and c.1432+5 G>T. These studies revealed that only the −KTS transcript is produced from mutant alleles, whereas both +KTS and −KTS transcripts are produced from the wild-type allele.^2^^,^^12^ Here, our *in vitro* assay revealed identical results for these 4 variants as well as for the other 2 rare variants (c.1432+1 G>A and c.1432+6 T>A) and a new variant (c.1432+3 G>T). Thus, it was confirmed that all intronic variants identified as being associated with FS to date disrupt the original splice donor site, resulting in production of only the −KTS transcript.

This study also focused on the production of +KTS/−KTS mRNA in renal tissue of the patients. Previous studies quantified the transcripts in patients by using a semiquantitative method based on agarose gel electrophoresis to determine the ratio of +KTS/−KTS transcripts, but the reported ratios show wide variation (Table 1). We quantified this ratio by RNA sequencing, revealing a +KTS/−KTS ratio of 1:0.85 in normal kidney and 1:3.2 to 3.5 in patients’ samples. Considering the ratio of +KTS/−KTS in a normal kidney, it was revealed that the +KTS transcript was not produced at equivalent levels for all analyzed variants. Although the performance of RNA sequencing for only 1 sample in each variant prevented us from examining the differences statistically, together with the *in vitro* assay, we concluded that all variants causing FS show the same splicing pattern.

In research on rare inherited diseases, large-scale clinical data on the disease including the genotype-phenotype correlation are vitally important for both clinicians and patients to predict the clinical course or provide genetic counseling. To date, no large-scale investigations on the renal prognosis of patients with FS have been performed. As described in this report, a systematic literature review of 126 cases revealed that the median age of ESKD onset among FS patients is 16 years and that there is no significant difference in the median renal survival period between those with the 2 major *WT1* variants in FS: c.1432+4 C>T and c.1432+5 G>A. This is compatible with our *in vitro* and *in vivo* mRNA study. All of these results led us to conclude that genotype does not influence renal prognosis in FS.

In a previous report, 65 cases of FS were summarized together with clinical descriptions of the patients’ external genitalia and sex chromosomes, and the ratios of patients with female external genitalia and XY chromosomes were calculated as 0.77 (17/22) for the c.1432+4 C>T variant and 0.93 (27/29) for the c.1432+5 G>A variant (*P* = 0.10, χ^2^ test).^28^ Consistent with this, our meta-analysis of data from 83 patients showed no significant difference in the ratio of these patients between variants c.1432+4 C>T and c.1432+5 G>A (*P* = 0.66, χ^2^ test).

Our study also focused on the influence of sex chromosome karyotypes and sexual development on renal prognosis. Sex differences in phenotype have been well established for various kidney diseases. In general, women are protected against renal diseases compared with men through estrogen’s inhibition and androgen’s activation of the renin-angiotensin system.^59^ Therefore, women show a lower risk of CKD progression than men.^60^ In addition, the renal prognosis in women with autosomal dominant polycystic kidney disease is better than that in men.^61^ However, women do not always have a lower incidence or severity of renal diseases; for example, women have been reported to have a higher risk of diabetic kidney disease.^62^ In this study, the median renal survival period tended to be longer in patients with a sex chromosome pattern of XX than in those with XY, although the difference was not statistically significant (29 vs. 16 years, *P* = 0.09, log-rank test). In addition, the renal prognosis in patients with XY chromosomes was not influenced by the presence of DSD (the median age of developing ESKD was 16 in DSD+ vs. 15 in DSD−, *P* = 0.84, log-rank test). Taking these findings together, differences in sex chromosome karyotypes and sexual development are not considered to influence the renal prognosis in FS.

Our study has some limitations. First, our analysis of the genotype-phenotype correlation is based on a retrospective systematic review. Thus, we could not collect clinical information regarding treatment or factors that might worsen renal function. Second, some of the reports that we analyzed in this study only focused on renal manifestations, with no detailed description of the sexual phenotype or tumorigenesis. The abnormalities of external genitalia seen in FS range from obvious discrepancies between the sex chromosome karyotypes and external genitalia to more subtle abnormalities. Therefore, the present study model may not be sufficient to clarify the correlation between renal genotype and the phenotype of sexual development in detail. However, we believe that our findings on the genotype and renal phenotype correlation in FS, which is the primary focus of this study, are important for clinicians, patients, and their families. We also conducted *in vivo* mRNA analysis only for urine-derived cell or kidney samples and not for other tissues such as gonads and blood samples. Therefore, we cannot assess the consistency of transcripts among different tissues. We also could not assess the difference in the +KTS/−KTS ratio between males and females. Finally, we did not conduct a sample size calculation because FS is a rare disease and we could only include all previous reported cases with sufficient clinical data. Therefore, the current results might have been affected by the small sample size.

In conclusion, we have confirmed that all variants, including the newly identified c.1432+3 G>T variant, result in the same abnormal splicing pattern as revealed by *in vitro* and *in vivo* transcript analyses. In addition, a large-scale meta-analysis suggested that renal prognosis was not influenced by the location of intron 9 variants, sex chromosomes karyotypes, or DSD. We believe that this study broadens our understanding of the genetic and clinical characteristics of FS.

## Disclosure

All the authors declared no competing interests.

## Supplementary Material

Supplementary File (PDF)

**Figure S1.** Comparison of the results of each patient in targeted RNA sequencing.

**Table S1.** Comparison of renal prognosis in patients with Frasier syndrome between different patterns of sex chromosomes and phenotypes of external genitalia.

**Table S2.** Prevalence of gonadal tumor in patients with Frasier syndrome depending on sex chromosomes karyotypes and phenotype of external genitalia.
